# The Interaction Between Dietary Fructose and Gut Microbiota in Hyperuricemia and Gout

**DOI:** 10.3389/fnut.2022.890730

**Published:** 2022-06-22

**Authors:** Xin-yu Fang, Liang-wei Qi, Hai-feng Chen, Peng Gao, Qin Zhang, Rui-xue Leng, Yin-guang Fan, Bao-zhu Li, Hai-feng Pan, Dong-qing Ye

**Affiliations:** ^1^Department of Epidemiology and Health Statistics, School of Public Health, Anhui Medical University, Hefei, China; ^2^Inflammation and Immune Mediated Diseases Laboratory of Anhui, Hefei, China

**Keywords:** fructose, gut microbiota, hyperuricemia, gout, interaction

## Abstract

With the worldwide epidemics of hyperuricemia and associated gout, the diseases with purine metabolic disorders have become a serious threat to human public health. Accumulating evidence has shown that they have been linked to increased consumption of fructose in humans, we hereby made a timely review on the roles of fructose intake and the gut microbiota in regulating purine metabolism, together with the potential mechanisms by which excessive fructose intake contributes to hyperuricemia and gout. To this end, we focus on the understanding of the interaction between a fructose-rich diet and the gut microbiota in hyperuricemia and gout to seek for safe, cheap, and side-effect-free clinical interventions. Furthermore, fructose intake recommendations for hyperuricemia and gout patients, as well as the variety of probiotics and prebiotics with uric acid-lowering effects targeting the intestinal tract are also summarized to provide reference and guidance for the further research.

## Introduction

In recent years, the prevalence and incidence of hyperuricemia and gout is increasing, and the age of onset has shifted to an earlier life (1–3). The rate of hyperuricemia prevalence more than doubled between the 1960s and the 1990s and continued to increase steadily afterwards until at least 2016 (3, 4), reaching to ~21%. As a result, between 1997–2012, the global gout prevalence rate increased from 1.5 to 2.5% (5). The increasing incidence of hyperuricemia and its associated co-morbidities (e.g., gout, type 2 diabetes, and cardiovascular disease) in the general population worldwide has greatly increased the public health burden on society (6). As a major public health issue, their need for clinical interventions has attracted significant interest. It is well-known that the consumption of sweeteners, mostly fructose, has increased dramatically after the Industrial Revolution, leading to a dietary shift in the world population (7). Given fructose consumption can stimulate the catabolism of adenine nucleotides to increase uric acid (UA) levels, the association between increased intake of fructose in the dietary profile and the risk of hyperuricemia and gout has been noted and confirmed by many studies, although some studies have inconsistent results (8–10). Besides, since ~25% of UA is excreted into the gut and further metabolized by gut bacteria, the gut microbiota nowadays has become a new target to understand the pathogenesis of hyperuricemia and gout (11). As diet shifts are important factors in the formation and modification of gut microbiota, which plays a key role in drug therapy, understanding the interactive roles of fructose and gut microbiota in hyperuricemia and gout are imperative to seek safe and effective clinical application. Therefore, this review provides an overview of the relationship among fructose intake, gut microbiota metabolism, and hyperuricemia or gout, as well as further discussion of their connective mechanisms. Moreover, the potential role of probiotics, prebiotics and fructose intake recommendations in the prevention and management of hyperuricemia or gout are further summarized.

## Manuscript Formatting

### Cause and Comorbidities of Hyperuricemia and Gout

The progression of hyperuricemia and gout includes four pathophysiological stages: the development of hyperuricemia, the deposition of monosodium urate crystals, gout flares caused by the acute inflammation to deposited crystals, and advanced disease featured with tophi (11).

#### Hyperuricemia

The hallmark of gout is the concentration of serum uric acid (SUA), whose solubility threshold is well-known to be affected by temperature, pH, and sodium concentration (11). In general, the definition of hyperuricemia is established when the concentration of SUA reachs 6.8 mg/dl (416 mmol/l), which is equivalent to the solubility threshold of urate at pH of 7.4 and temperature of 37°C (11, 12) in human. However, the definition of hyperuricemia varies with age and gender (13). It has been defined as SUA>7.0 mg/dL (420 mmol/l) in men; >6.0 mg/dL (360 mmol/l) in women; and >5.5 mg/dL (330 mmol/l) in children and adolescents (13). Uric acid (UA) is the final catabolic product of human exogenous and endogenous purine nucleotide metabolism (14). In most mammals, UA produced by metabolism can be degraded by uricase into 5-hydroxyisoourate and allantoin to be eliminated from the body (15). However, humans and great apes who lack uricase because of its mutation or inactivation during evolution, have higher circulating urate concentrations (16). Hyperuricemia can result from excessive purine intake, purine metabolism in the body, or mainly by renal and intestinal underexcretion (17), which is an important step in the development of gout. However, only about 36% of patients with hyperuricemia will eventually develop into patients with gout, whereas most remain in a state of asymptomatic hyperuricemia (18, 19). Therefore, hyperuricemia is generally considered asymptomatic in the absence of inflammatory episodes (gout flares) caused by monosodium urate (MSU) crystals.

Asymptomatic hyperuricemia is highly prevalent worldwide, ranging from 2.6 to 36% in different populations, and it has been increasing for decades (4, 20). Epidemiological and experimental data obtained over the past few decades show solid correlations between asymptomatic hyperuricemia and hypertension (HTN), renal disease, cardiovascular events, type 2 diabetes (T2DM), and other metabolic diseases. A meta-analysis of 55,607 patients found a dose-dependent relationship between serum urate and HTN; for every 1 mg/dl increase in SUA, the relative risk of HTN increased 1.13 times (21). The meta-analysis of Li et al. that reviewed 190,718 participants in 13 observational cohort studies inferred that elevated serum uric acid levels are associated with the onset of chronic kidney disease [OR = 2.35 (1.59–3.46)] (22). Krishnan et al. conducted a follow-up study of 5,012 diabetes-free participants aged 18–30 years for 15 years and reported that asymptomatic hyperuricemia is an independent risk factor for T2DM and insulin resistance [HR = 1.87 (1.33–2.62) and 1.36 (1.23–1.51), respectively] (23). Two large-scale meta-analyses that explored the correlation between the incidence of T2DM and serum urate also found similar results, suggesting that for every 1 mg/dl increase in serum urate, the combined relative risk of T2DM increases by 6–11% (24, 25). Another meta-analysis includes six studies of 5,686 patients with acute myocardial infarction showed that patients with hyperuricemia are more likely to have major adverse cardiac events [RR = 3.44 (2.33–5.08)] and in-hospital mortality [RR = 2.10 (1.03–4.26)] (26). Cell culture and animal studies further prove that hyperuricemia may be related to renal failure, hypertension, and metabolic syndrome (15, 27). Therefore, the researchers then conducted Mendelian randomized studies to confirm the causal association; however, the inconsistent results led to the causal link among hyperuricemia and kidney disease, hypertension, diabetes, or other metabolic diseases remains controversial (28–30). Indeed, the time interval from the onset of hyperuricemia to that of cardiovascular and renal comorbidities is long, making it unclear whether hyperuricemia is a risk marker for non-gout diseases or independent causal factors. In addition, although hyperuricemia is earlier than comorbidities, reverse causality cannot be ruled out. Moreover, the population with heterogeneous and environmental factors are not considered in the Mendelian randomization study. Also, there is a lack of understanding of the potential impact of genetic polymorphisms on the urate-related biological mechanisms.

The causality between hyperuricemia and comorbidities such as kidney disease, cardiovascular events, and diabetes is still not entirely resolved. However, given the rising prevalence of asymptomatic hyperuricemia, as well as metabolic and renal diseases, it is vital to explore the pathogenesis of hyperuricemia, especially the risk factors that can be manipulated.

#### Gout Flares

Gout flare is the most significant and unique manifestation of gout, leading to the extreme pain of synovitis in the joints and surrounding areas, soft tissues and other organs bounded by monosodium urate (MSU) crystal deposits (31). Theoretically, once the SUA level reaches the solubility threshold, it can precipitate as needle-like crystals and cause inflammation. In fact, even in hyperuricemia patients whose SUA concertation is higher than 10 mg/dL, only 50% of them had a gout flare over 15 years (32). Therefore, it is suggested that hyperuricemia is necessary but insufficient to induce gout, and additional molecular/gene factors can affect the formation of MSU. Approximately 25% of patients with hyperuricemia have MSU, and it often occurs in the first metatarsophalangeal joint (19).

MSU crystals are a potential trigger for inflammation, but the mechanism of how MSU crystals induce inflammatory response has not been fully elucidated. Activation of PYD domains-containing protein 3 (NLRP3) inflammasomes in macrophages and monocytes by MSU is recognized as central to the initiation of the gout flare (33). NLRP3 inflammasome is a cytoplasmic protein complex involved in the maturation and expression of pro-inflammatory cytokines such as IL-1β, IL-33, and IL-18 (34). In the last decade, trials of inflammation-modifying treatments, such as IL-1β inhibition, have been tested as a treatment for gout with successful outcomes (35). The activation of NLRP3 inflammasome has been described as a dual-signal initiation process. The first signal stimulates NF-κB through TLR4 and TLR2 and synthesizes pro-IL-1β and inflammasome components (36). The second activation signal is more specific and is mediated by MSU crystal, causing the assembly of inflammasomes. The oligomerization of NLRP3 inflammasomes leads to the activation of Caspase-1, which proteolyzes pro-IL-1β into biologically active IL-1β. Then, IL-1β interacts with IL-1β receptors, triggering a downstream signal cascade including pro-inflammatory cytokines and chemokines, causing the recruitment of neutrophils and other cells to the site of crystal deposition (37).

Acute gout attacks, especially in the early stages of the disease, are self-limiting by nature (38). Current research shows that there are multiple mechanisms involved in the spontaneous resolution of acute gout, including protective protein-encapsulated crystals, and changes in the expression balance of pro-inflammatory and anti-inflammatory factors when the cell population in the inflammatory joint changes (39). After years of acute intermittent gout, advanced gout characterized by tophi, chronic gouty synovitis, and structural damage in joints usually occurs. The cytokines, chemokines, proteases and oxidants involved in acute inflammation induced by UA can cause chronic inflammation, leading to chronic synovitis, cartilage loss and bone erosion (40).

In addition to the excruciating arthritic pain and premature death, gout is associated with a high frequency of comorbidities, such as insulin resistance syndrome, hypertension, obesity, nephropathy, and cardiovascular disorders. The prevalence of comorbidities with increasing duration of gout is associated with all components of metabolic syndrome (41). Prospective studies have consistently shown that patients with hypertension have an increased risk of gout (42–44). According to the third NHANES (spelt out its whole names), abdominal obesity and T2D prevalence were found higher in gout patients (respectively 62.9 and 33.1%) than that in non-gout patients (respectively 35.3 and 10.8%) (45). Moreover, in prospective studies, gout also increases the risk of T2D, and diabetes reduces the risk of gout (46, 47), which can be partly explained by the increased excretion of urate in urine of diabetic patients (48). In addition, the prevalence of CKD in stage 3 or above in gout patients is estimated to be 24% and (49) gout prevalence was found to increase from 16.0 to 35.6% for CKD patients (50). Moreover, various heart diseases are independently associated with gout, which lead to increased frequency of cardiovascular deaths (51, 52).

Therefore, comorbidities of gout must be given special attention for the disease because they may contribute to the critical prognosis of gout patients and complicate the management of gout. Furthermore, deep understanding of the underlying molecular mechanism of gout flare is incredibly crucial.

### Bridging Fructose Intake and Hyperuricemia or Gout

Increasing our understanding of the correlation between fructose intake and hyperuricemia is important because fructose consumption has risen dramatically in the last 40 years and constitutes a large proportion of the daily caloric intake in the modern diet (7). The total fructose consumption rate in the US increased by 33% from 1978 to 2004, and the most significant increase was among people aged 19–22 years old with a mean increase in added fructose intake of 33 and 30 g/d, respectively (53). The overall estimated mean total fructose comprising energy intake also increased, from 8.1% in 1978 to 9.1% in 2004 (53). The consumption of added sugars (half of which is likely fructose) gradually decreased after that but remained at 17% of total energy intake, which is still far above the recommended upper limit of 10% (54). In the non-US populations, fructose consumption also showed a similar trend. In 1968–2007, sugar consumption rose gradually from 56 to 65 g/d, and the most impressive rise was in Asia from 30 to 45 g/d with a 50% increase (55). In recent years, the intake of added sugars has also declined or stabilized, but the amounts consumed still represents 10%-13% of total calorie intake (56). Moreover, intake of added sugars (up to 19% of total energy) of school-age children and adolescents was higher than that of younger children or adults (56).

Interestingly, the prevalence of gout and hyperuricemia have been found a similar trend during the overlapping period. In the US, the prevalence of gout and hyperuricemia was more than doubled between the 1960s and the 1990s, and then continued to increase steadily, at least until 2007–2008, and began to stabilize for the next 10 years (4). Studies in other countries over a similar period have shown a steady increase in the prevalence of gout and hyperuricemia. According to the nationally representative New Zealand Health Survey, the overall prevalence of gout among those aged 15 years or older nearly doubled from 1.6% in 2011–2012 to 2.9% in 2015–2016 (4). Using administrative health declarations, another study in the Canadian population found that the prevalence of gout in the overall population rose from 2.4% in 2000 to 3.8% in 2012 (57). In addition, a study based on the general practice population in the UK also found an increase from 2.03% in 2007 to 2.49% in 2012 (5). In line with the data from the western countries, a systematic review showed that the prevalence of gout in China from 2000 to 2014 has risen to 1.1% (3).

It's not surprising to found that a large number of epidemiological studies and interventive experiments have focused on exploring the association between fructose intake and gout or hyperuricemia; however, inconsistent results were obtained. Due to the lack of an effective calculation method used to determine total fructose intake, most epidemiological articles on the association between dietary fructose and gout mainly focus on fructose-containing foods and gout. As we all know, food sources containing fructose sugar include non-alcoholic beverages (SSBs), fruits and fruit products, dairy products, cereals and cereal products, as well as candies, chocolates, and desserts (58). Since 1970, high fructose corn syrup (HFCS) has become the most popular sucrose substitute in most soft drinks and fruit drinks, baked goods, canned fruits, jams and jellies, and dairy products due to its low price and higher sweetness (59). As a result, soft drink consumption has increased dramatically. For instance, from 1977 to 1997, American adults' consumption of soft drinks increased by 61%, and sweetened soft drinks became the largest single food source in the American diet (60). Naturally, the association between SSBs and gout or hyperuricemia has received the greatest attention and has been most studied. Two large prospective studies containing a total of 125,299 participants over an average of 17 years of follow-up have identified a significant association between SSB consumption and increased risk of gout [RR = 1.62 (1.28, 2.03)] (61, 62). Another systematic review and meta-analysis (58) assessed essential food sources of fructose-containing sugars with incident gout. The results have documented that there was moderate certainty of evidence that SSB intake was associated with a 208% increase in gout incident; fruit juice has a relatively low rate of gout, which is related to a 77% increase in gout; and that fruit intake was not associated with gout incident (58).

Through a 10-year follow-up observation of the cohort from 2008 to 2018, the actual age of onset of gout in China was 4.14 years younger, and sugar-sweetened soft drinks was found as an independent predictor of early-onset gout (63). Additionally, many studies have shown that high fructose or fructose-containing food intake is linked to increases in SUA concentration, which leading to the development of gout. According to the NHANES 2001-02 database, Gao et al. found that men's intake of more added sugar or sugar-sweetened beverages was associated with higher plasma uric acid concentration, while women did not; Intake of fruit juice, which contains a lot of naturally occurring sugars, however, was not related to plasma uric acid (64). Besides, two meta-analyses have also disclosed that SSB intake was associated with raised SUA (65, 66). In contrast, using data of added sugar disappearance and naturally-occurring sugar contents to assign conversion factors to food groups to determine individual fructose intake, NHANES 1999-2004 survey failed to disclosed the relationship between dietary fructose intake and a higher risk of hyperuricemia (67). To some extent, several reasons may appear to account for these inconsistences. Firstly, fruits are demonstrated not to increase the risk for gout or hyperuricemia (9). The nutrients present in the fruits such as vitamin C, epicatechin, flavonols, potassium and fiber can change the effects of fructose and uric acid. Furthermore, people who consume a lot of fruits usually reduce their intake of refined sugar, so total fructose intake may be low. Secondly, the amount of fructose contained in the fruits and vegetables was not separately analyzed in the NHANES 1999–2004 survey. Lastly, the calculation method used to determine total fructose intake in the survey is somehow lack of accuracy because the data for naturally-occurring sucrose contents for most foods are not available.

Besides, a series of intervention trials exploring the relationship between fructose administration and serum uric acid concentration produced inconsistence results. MacDonald et al. noticed that within 90 min after 9 healthy young men drank a pure fructose beverage (1 g/kg body weight), the serum uric acid concentration increased by about 10% (68). Consistently, Emmerson demonstrated that in three healthy men, after ingesting 250–290 g/d fructose, the serum uric acid concentration was 8–41% higher than that of glucose (69). Likewise, another two fructose infusion studies (0.5 g/kg BW, in 8 healthy young men and 4 gout patients) also observed elevated SUA levels (70). However, other studies failed to repeat these results. For example, in the Turku Sugar Study in Finland, 35 healthy subjects ingested added fructose at a level of 2.1 kg/month (about 70 g/d) for 22 consecutive months, and there was no significant increase in serum uric acid concentration or uric acid excretion (71). Other studies also reported that long-term (1–6 months) intake of added fructose (11–18% of daily calories) has no effect on the serum uric acid concentration of diabetic patients (72–76).

Along the same line, Crapo et al. (77). observed that in 11 normal participants, 24% of daily calories given to fructose for 2 weeks did not increase SUA concentration and urine excretion. Narins and his colleagues reported that adding fructose at a fructose intake of 100 g/day for 5 days did not lead to an increase in serum uric acid concentration in healthy adults (70). In addition, Turner et al. have found that there was no increase in serum uric acid after drinking beverages containing 90–154 g of fructose in 6 patients with high triglycerides (78). Similarly, Curreri's study showed that in 20 young men, 100 g of fructose infusion did not cause an increase in SUA (79). For the inconsistent results observed in the intervention trial, one possible explanation may be the difference between the subjects and the study protocol. Another important point is that comparison with co-intake of other carbohydrates (such as glucose), and with the intake of fructose alone may have very different absorption, metabolism and physiological effects. For example, Riby et al. documented the synergistic effect of glucose for fructose malabsorption (80). In the daily diet, fructose is rarely taken alone. In this study, dietary fructose intake is found to be related to other sugars, and the Pearson and Spearman correlation coefficient between fructose intake and total sugar intake is 0.94 (*P* < 0.001). In summary, despite the inconsistent results in the literatures, most studies have shown that high fructose intake can lead to hyperuricemia. Therefore, the mechanism of the influence of fructose intake on SUA concentration and the occurrence of hyperuricemia deserves more attention.

### Role of Fructose and Gut Microbiota in Hyperuricemia and Gout

#### Fructose Metabolism and Uric Acid Production

The essence of hyperuricemia is the result of the imbalance between UA production and excretion. Interestingly, fructose is the only common ingested carbohydrate that produces uric acid during metabolism (81). Fructose is a kind of hexose, and its chemical formula C_6_H_12_O_6_ is the same as glucose. It differs from glucose in that it has a ketone group at position 2 of the carbon chain, while there is an aldehyde group at position 1 of the glucose carbon chain. In solution, it can exist in the form of α or β pyranoside and furanoside rings. The content of fructose and glucose in ordinary sucrose is generally in a 1:1 ratio. High fructose corn syrup also contains a mixture of fructose and glucose. The outcomes of systemic fructose homeostasis are mainly 2-folds: intestinal absorption and clearance, the latter of which is generally considered to be mainly mediated by the liver (~55–71%) and the kidney (<20%) (82).

**Figure 1:**
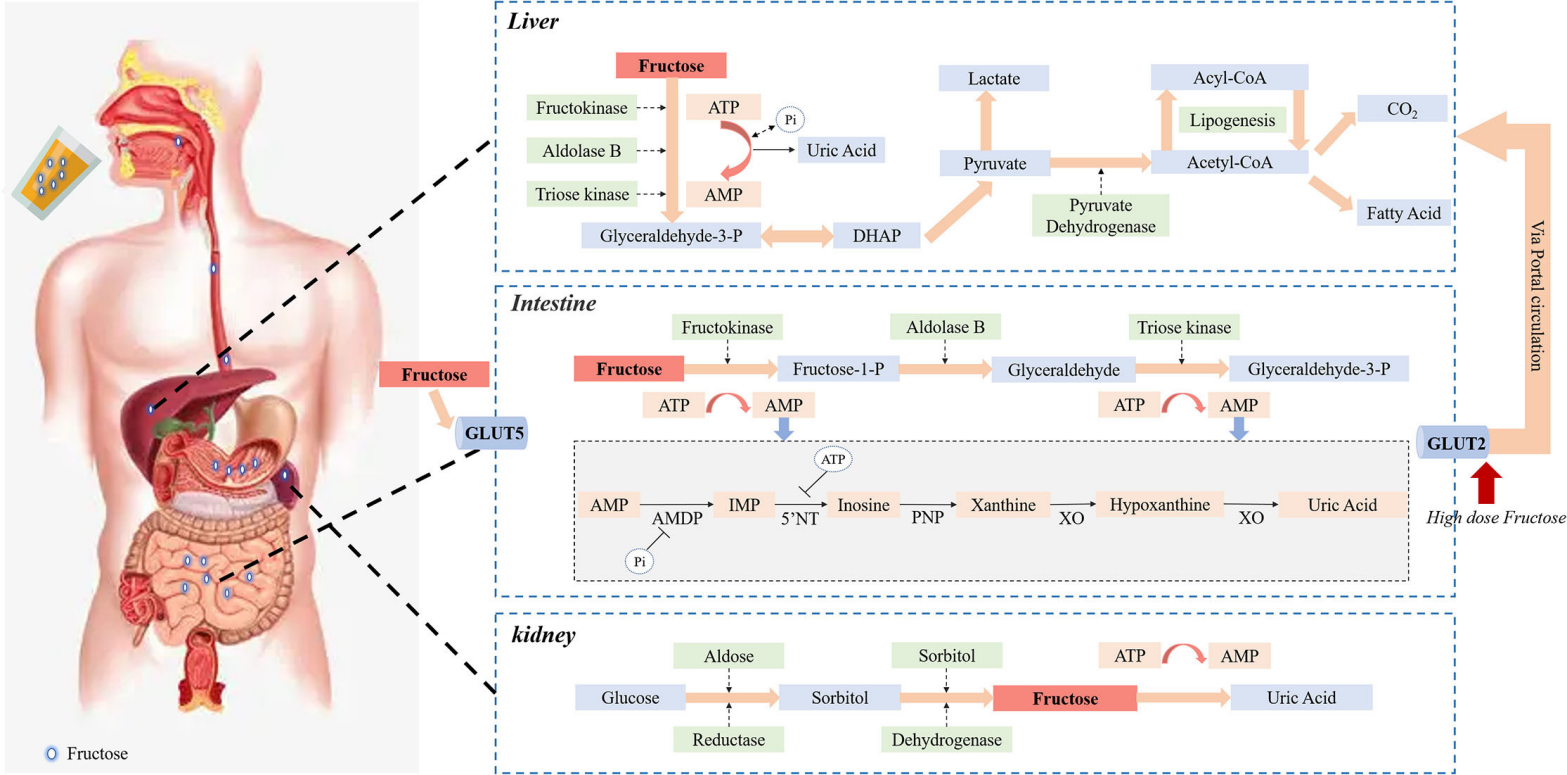
Fructose metabolism in the body. The fructose ingested by the body will be first transported to the enterocyte via GLUT5, and then absorbed and metabolized in an insulin-independent manner. Through all the processes of metabolism in the small intestine, uric acid is produced. The rapid phosphorylation of fructose into fructose 1-phosphate requires ATP to provide a phosphate molecule, which reduces intracellular levels of ATP, GTP, and phosphate, leading to the accumulation of AMP and activation of AMP deaminase. AMP generates IMP under the action of AMP deaminase. IMP levels are elevated, which is metabolized with GMP to produce uric acid. Uric acid, in turn, inhibits AMP-activated protein kinases, which promotes more AMP metabolism through AMP deaminase, resulting in more uric acid production. The extent to which fructose enters the liver through the small intestine depends on the amount of fructose ingested. Fructose metabolism appears to be a saturation process in the intestinal, with only high doses of dietary fructose spreading to the portal circulation through the transporter GLUT2 and further extracted by the liver. Fructose and eventual metabolism in the liver produce pyruvate and acetyl-CoA, leading to adipogenesis, and the metabolic processes accompanied by rapid depletion of intracellular ATP and Pi and the production of corresponding uric acid. Renal proximal straight tubule expression of fructokinase and aldolase B is the main site of fructose metabolism under physiological conditions, continuous intake of large amounts of fructose leads to renal metabolism exceeding the threshold, resulting in a large amount of ATP depletion and inflammatory response, and finally a large amount of uric acid production and tubular damage. In addition, endogenous fructose obtained by the polyol pathway in the kidneys may be another potential mechanism for causing kidney damage and uric acid production.

Figure 1 has depicted the details of fructose metabolism in the body. Once the body directly ingests pure fructose or HFCS, or fructose is produced from the digestion of sucrose on the brush border membrane, it will be transported from the lumen to the enterocyte via a specific fructose transporter GLUT5, which is located at the apical pole of the enterocyte. Subsequently, fructose is firstly absorbed and metabolized by the small intestine in an insulin-independent manner (83). In the first step, fructose is rapidly phosphorylated under the action of fructokinase [also known as ketohexokinase (KHK)] to form fructose-1-phosphate. This process requires ATP to provide a molecule of phosphate to be converted into ADP. In the second step, fructose-1-phosphate is decomposed by aldolase B into glyceraldehyde and dihydroxyacetone phosphate. In the final step, trikinase catalyzes the phosphorylation of glyceraldehyde by ATP to form glyceraldehyde- 3-phosphate (83). In this process, the rapid phosphorylation of fructose into fructose 1-phosphate reduces the intracellular levels of ATP, GTP and phosphate, leading to the accumulation of AMP (84). The decrease of intracellular GTP and phosphate activates AMP deaminase, and the accumulation of fructose 1-phosphate has did the same effect. AMP generates IMP under the action of AMP deaminase. IMP levels rise, and it is metabolized along with GMP to generate uric acid (85). In turn, uric acid inhibits AMP-activated protein kinase, thereby promoting more AMP metabolism by AMP deaminase. Influencing aldose reductase and increasing the expression and activity of fructokinase at the same time can stimulate fructose production further (86–88). In addition, the accumulation of IMP, GDP and GMP inhibits aldolase B, further enhances fructokinase activity and stimulates ATP conversion (84). As uric acid accumulates, xanthine oxidase is inhibited, but it may be at the cost of continuous activation upstream of the AMP deaminase pathway (89).

After fructose absorption and clearance, the remaining fructose inside the enterocyte diffuses into the portal circulation via a transport GLUT2 located at the basolateral pole of the enterocyte (90) and is further extracted by the liver rapidly and efficiently. Considering that the liver presents a high level of fructose catabolism enzymes (like KHK) expression and the sensitivity to fructose, it has generally been assumed to be the leading site of fructose metabolism (91). However, this notion has recently been challenged. Researchers (92, 93) have discovered that the small intestine shields the liver from fructose exposure. Most of the fructose intake is metabolized through the small intestine of mice. Using isotope tracers and mass spectrometry in mice, it was surprising that most dietary fructose has been converted into glucose and various organic acids in the portal vein, which connects the small intestine to the liver (93). The extent to which unmetabolized fructose enters the liver through the small intestine depends on the absorption and clearance rate of fructose in the small intestine. When ingesting low-dose fructose (< 0.5 g kg^−1^), about 90% of fructose phosphorylation occurs in the jejunum, duodenum, or ileum. In contrast, intake of high doses of fructose (≥1 g kg-1) will saturate the absorption and catabolism of fructose in the small intestine, causing fructose to overflow into the liver (>30%) (92, 93). Namely, intestinal fructose metabolism seems to be a saturated process, allowing only high doses of dietary fructose to enter the liver. Most of the entered fructose is rapidly phosphorylated in the liver, and under the action of three key enzymes, glyceraldehyde 3-phosphate, an intermediate product of the glycolysis pathway, is generated (92). After that, the metabolic pathways of fructose and glucose in the liver are qualitatively similar and finally metabolized to produce pyruvate and acetyl-CoA, leading to lipogenesis (82). Another consequence of the fructolysis is the rapid depletion of intracellular ATP and Pi and the production of corresponding uric acid.

Another important organ for fructose metabolism is the kidney. The proximal straight tubule is the main site of fructose metabolism under physiological conditions. There, the glucose transporter 5 expressed at the top of the cell membrane mediates the absorption of fructose in the urine, which is then metabolized by fructose kinase in the cytoplasm. However, fructose metabolism is not limited to the proximal straight tubules; the proximal curved tubules have also been recorded. In fact, the proximal tubules express fructokinase and aldolase B, the latter being easily induced (94). On the contrary, continuous intake of a large amount of fructose may cause the physiological mechanism of the kidney to metabolize fructose to exceed the threshold. As a result, a large amount of ATP consumption and inflammatory reactions occur, and ultimately lead to a large amount of uric acid production and renal tubular damage (95). In addition to exogenous fructose, fructose can be synthesized from glucose through the polyol pathway. Glucose is converted to fructose in two steps in the polyol pathway. Firstly, aldose reductase regulates the conversion of glucose to sorbitol. Subsequently, sorbitol dehydrogenase catalyzes the oxidation of sorbitol to fructose. The endogenous fructose obtained via the polyol pathway in the kidney may be a potential mechanism for causing kidney damage (96). In mice with acute ischemic kidney injury, an increase in the concentration of aldose reductase, sorbitol, and endogenous fructose was found in the renal cortex, indicating that the polyol pathway was significantly activated. Moreover, polyol pathway restriction can limit acute ischemic kidney injury and promote recovery after kidney injury (97).

Based on the metabolism of fructose, it is can be concluded that fructose intake could induce UA synthesis through increasing of ATP decomposition as a precursor of UA, and increased insulin level following fructose intake has also been shown to raise of urate reuptake and reduce the excretion of UA in kidney. More specifically, fructose is rapidly phosphorylated upon intake, which is thought to lead to ATP depletion and the subsequent AMP accumulation (98). And the lack of free phosphate leads to AMP convert to IMP, thus resulting in the production of UA and the amelioration of UA levels in the serum (98). High fructose levels and associated decrease in ATP in the body will increase the compensatory effect of purine nucleotide synthesis, which subsequently results in further overproduction of UA in the presence of additional fructose (99). In addition, high fructose levels induce hyperinsulinemia and insulin resistance, which may lead to further elevated circulating UA levels via reducing UA excretion (96).

#### Interaction Between Fructose-Rich Diet and Gut Microbiota in Gout or Hyperuricemia

Given that ~30% of UA was excreted by the gastrointestinal (GI) tract, it is not surprising that gout or hyperuricemia-related studies have focused on the analysis of gut microbiota in recent years. The GI tract provides one of the largest interfaces (250–400 m^2^) among the host, environmental factors, and antigens in the human body (100). Over 10^14^ microorganisms have been inhabiting the GI tract, which offers many benefits to the host through a series of physiological functions. However, there is potential for these mechanisms to be destroyed as a result of an altered microbial composition, known as dysbiosis or dysregulation, which are associated with a variety of diseases (101–103). As for hyperuricemia, the gut microbiota is known to be involved in the metabolism of purines and uric acid. For example, the key enzyme in the oxidative metabolism of purines, xanthine dehydrogenase, was proved to be secreted by the *Escherichia coli* group of human intestinal bacteria (104–106). *Proteus* bacteria can secrete xanthine dehydrogenase to convert purines into UA (104). *Lactobacillus* can reduce the absorption of purines in the intestine, prevent the increase levels of serum UA and further induce hyperuricemia (107). Common members of the human gut microbiota, *Lactobacillus* and *Pseudomonas*, are found to be active in the synthesis of key enzymes (such as uricase) in the catabolism of UA (108). Moreover, the study also found that various indigenous microorganisms in the human gut secrete uric acid transporters, thereby affecting the excretion of UA (109). Therefore, intestinal microbiota can potentially affect the production and excretion of UA, thereby participating in the pathogenesis of hyperuricemia or gout.

Indeed, recent sequencing studies have shown special changes in microbial gene richness and diversity in gout subjects when compared to healthy controls (110). The abundance of *Bacteroides caccae* and *B. xylanisolvens* was significantly enriched in gout subjects, while those of *Faecalibacterium prausnitzii* and *Bifidobacterium pseudocatenulatum* was reduced (110). Further metagenomes study also found that the abundances of *Prevotella, Fusobacterium*, and *Bacteroides* were increased in gout patients, whereasthat of *Enterobacteriaceae* and butyrate-producing species were decreased (111). Also, imbalanced intestinal flora was observed in the rat model of nephropathy induced by hyperuricemia using the 16S rRNA technology (112). *Flavobacterium, Myroides, Corynebacterium, Alcaligenaceae, Oligella* and other conditional pathogens increased greatly in the rat model group, whereas *Blautia* and *Roseburia*, and the SCFAs producing bacteria diminished significantly (112).

Diet is one of the well-known significant factors that impact the composition of gut microbiota. A high-fiber diet appears to be involved in the increased abundance of *Rominococcus bromii* (113) and depletion of *Frimicutes* /*Bacteroidetes* (F/B) ratio (114), leading to the high production of SCFAs and butyrate in the host (113, 114). Diet supplemented with high-fat in mice indicated an association with the enrichment in F/B ratio, *Ruminococcus* and *Rickenellaceae*, as well as thedepletion in *Lactobacillus, Bifidobacterium*, and *Prevotella* (115, 116). In addition, artificial sweeteners were demonstrated to have deleterious effects on the composition of the gut microbiota, resulting in the rise in *Fecal Bifidobacteria, Lactobacilli, propionate* and reduction in *Bifidobacteria, Lactobacilli*, and *Bacteroides* (117, 118). Diet supplemented with a high-fructose syrup (HFS) compared to a fruit-rich diet can induce significant differences in the composition of the microbiota, resulting in a lower abundance of *Firmicutes* and *Ruminococcus* and a higher *Bacteroidetes* abundance (119).

Collectively, these research results made us comes up with a new question, that is, how does diet affect the interaction between the human body and its gut microbiota? Unfortunately, there is a lack of evidence to investigate the impact of fructose-rich diets on gut microbiota and the subsequent effects of high-fructose diet-induced effects on gout/hyperuricemia. An animal study in Rats with intragastrical administration of a high-dose fructose to increase uric acid resulted in an increased abundance of *Lachnospira, Parasutterella, Marvinbryantia, and Blantia* (120). However, a cross-over intervention study of 26 healthy adults who consumed either orange juice (OJ) or caffeine-free cola in -between 3 meals per day within 2 weeks, have found that beverage did not induce microbial changes and the uric acid level was decreased in the OJ group (121). The result makes sense since the complex nutrients which may have a positive effect on gut microbiota and stimulate UA excretion do exist in the OJ. Therefore, further studies with large-sample and other experimental conditions (e.g., different sources and forms of fructose, various doses and durations) might be needed.

#### Possible Mechanisms

How does fructose-mediated microbiota population shift affect the onset of gout/ hyperuricemia? To elucidate this doubt, the prerequisite is to understand the host-gut microbiota metabolic interactions (100). The composition and activity of the gut microbiota are subject to complex interactions that depend on the host's genome, nutrition, and lifestyle. On the other hand, the metabolism and homeostasis of the resident microbiota are closely related to the health of the host.

Recent studies found that high-dose fructose intake with the capability of inducing the overproduction of plasm inflammatory cytokines, such as IL-6, TNF-α, MIP-2, and IL-1β, decreased the level of anti-inflammatory marker IL-10 in rats (120, 122). Furthermore, excessive fructose intake alters the composition of the intestinal microbiota and impairs intestinal barrier function via reduction expression of TJ proteins, thereby triggering the inflammatory process (123, 124). As a complex, multi-gene inherited auto-inflammatory disorder, innate inflammatory pathways deservedly played a crucial role in the pathogenesis of gout (125). IL-1β is considered to be the core of gout inflammation. By stimulating pro-inflammatory cytokines and chemokines and up-regulating adhesion molecules on endothelial cells, inflammatory cells (like neutrophils and monocytes) are recruited to the MSU crystal deposition site, ensuing a positive feedback loop of inflammation, leading to ongoing inflammation (126). MSU crystals can stimulate the recruited monocytes to produce TNF-α (127). TNF-α facilitates the activation of caspase-1 by ATP via its receptors TNFR1 and TNFR2, leading to the activation of the NLRP3 inflammasome, which leads to the severity of gout. In addition, TNF-α can also promote the synthesis of pro-IL-1β mRNA, thereby promoting the production of IL-1β (128, 129).

Interestingly, these inflammatory reactions, which play a key role in the pathogenesis of gout, are also important factors for shaping and maintaining the homeostasis of intestinal flora. For instance, NLRP3 knockout mice exhibit both quantity and composition alterations in the microbiota, including a significant decrease in the F/B ratio (130) and only detected members of the genera *Mycobacterium* and *Collinsella* (131). In addition, the trafficking of immune cells following the increased release of inflammatory factors has been widely confirmed to synergistically promote the disruption of the intestinal mucosal barrier, thereby affecting the homeostasis of the intestinal flora (132). In turn, inflammatory reactions are influenced by gut microbiota and its products. The absence of microbiota is correlated with decreased production of SCFAs, especially acetate, which is necessary for inflammasome assembly and IL-1β production, and it is dependent on the activation of GPR43 (133), whose signal transduction can regulate the production of ROS (134) following activation of the NLRP3 inflammasome (135). Another interesting study found that gut dysbacteriosis can increase intestinal permeability and promote the translocation of bacteria or bacterial products, such as lipopolysaccharide [LPS (136)]. High levels of serum LPS can induce chronic inflammation and increase the risk of hyperuricemia (137). The high levels of LPS sustained in gout are positively related to the abundance of Gram-negative bacteria in the intestine, especially Proteobacteria (138). In addition, LPS is also a metabolite of the intestinal microbiota. Abnormal levels of LPS in blood circulation are usually accompanied by an increase in XO activity, which is an important enzyme in purine oxidation metabolism Furthermore, a recent study has stated that there were consistent associations between gut bacteria and immune cell dynamics, especially in the taxa of *Faecalibacterium, Ruminococcus 2*, and *Akkermansia* (139).

Additionally, the homeostasis of intestinal microbes will lead to changes in the metabolome, which are mainly manifested as the up-regulation of glucose, acetate, succinate and some amino acids, and the down-regulation of α- ketoisocaproate, phenylalanine, valine and citrulline (140). These metabolites are shown to be related to UA excretion, purine metabolism and inflammation. For example, acetate, succinate and glucose, materials that are involved in energy metabolism, can provide energy for intestinal epithelial cells, promote UA excretion and thereby relieve hyperuricemia (141). Glycine and aspartate are associated with the biosynthesis of purine nucleosides, and their abnormal expression leads to disorders of purine metabolism in patients with gout, thereby promoting the risk of disease (142). Besides, researches have shown that indigenous microbes in the human gut can secret key transporters to mediate UA absorption (109, 143–145). These transporters include *ABCG2, SLC2A9, SLC16A9, SLC17A4, SLC17A1, SLC17A3, SLC22A11, SLC22A12*, and *SLC16A9*, which in turn affect the absorption and metabolism of fructose. When the absorption of fructose in the intestine changes, the concentration of fructose in the lumen will also change, thereby affecting the ecology of intestinal microbes (90).

Moreover, the concept of oxygen metabolism and oxygen barrier in shaping the composition of the gut microbiota has recently been proposed (146). High fructose intake will increase the permeability of intestinal epithelial cells (147), resulting in increased oxygen levels in the lumen and following the destroy of living environment of epithelial anaerobes, which can further be facultative anaerobes or potential Aerobic bacteria provide the advantage of ecological selection, making them more competitive when expanding (148). For example, pathogenic bacteria such as Salmonella undergo aerobic proliferation under the destruction of anaerobic bacteria (147). These pieces of evidence support that oxygen levels can act as a shaper of the host's regulation of the gut microbiota. Overall, while understanding of the relationship among the microbiome, fructose, and gout or hyperuricemia has increased, more data are necessary to draw more concise and causal conclusions.

### Management of Gout or Hyperuricemia by Fructose and Gut Microbiota

The central strategy for effective, long-term management for gout or hyperuricemia is continuous urate-lowering therapy (ULT) to decrease the SUA level (12). However, the low efficacy of or poor adherence to ULT has already placed a long-term disease burden on society (149). Evidence from a systematic review (150) that accumulated 18 observational studies from 1974 to 2016 showed that the non-adherence rate is as high as 21.5–82.6% and the non-persistence rate (with a pause of at least 30 days during treatment) even reaches 54–87%. Thus, seeking safe, effective, and side-effect-free ways to prevent or treat HUA is of great significance in the management of gout or hyperuricemia.

Recently, the use of probiotics and prebiotics as alternative methods to treat hyperuricemia or gout without causing adverse side effects is being evaluated (Table 1). Probiotics are substances that are composed of yeast or live bacteria and have multiple health effects. They are considered potential natural treatments due to their beneficial effects in regulating the intestinal microbiota (158). Some evidence suggests that probiotic supplements can significantly prevent the accumulation of UA (159–161). Indeed, a diet rich in probiotics was shown to maintain the balance of the intestinal flora caused by HUA, increase the abundance of *bifidobacteria* and *lactobacilli*, and reduce serum levels of UA and LPS and XO activity (162). Moreover, some prebiotics that can exert an anti-HUA effect via the alteration of gut microbiota have been studied. For example, the reduction of *Bacteroides* and *Bifidobacteria* found in the HUA rat model can be subsequently restored by supplementing with tuna oligopeptides (TMOP). Furthermore, after transplanting the fecal microbiota of TMOP-treated mice, the phenotypes of hyperuricemia and renal inflammation were attenuated, indicating that the beneficial effects of TMOP on HUA are at least partially mediated by intestinal microbes (163). In addition, prebiotics can also reduce chronic inflammation in the host by regulating the intestinal microbiota, thereby alleviating hyperuricemia. For instance, sunflower head enzyme hydrolysate (SHEH) can alleviate HUA by promoting the recovery of the disturbance of intestinal flora composition and reducing the level of circulatory system LPS (164). Dendrobium officinale leaf extract can inhibit UA through the intestinal microbiota-LPS-TLRs/NF-κB axis to effectively treat HUA (165). Similarly, a growing body of studies from China has shown that many herbs and phytochemicals such as six kinds of dendrobium officinale, stevia residue extract, chicory, mangiferin and tigogenin, can play a prebiotic role in the prevention and treatment of HUA (166–170).

**Table 1:** Probiotics and prebiotics in management of hyperuricemia and gout.

**Study year**	**Name**	**Study type**	**Mechanism**	**References**
2020	Inulin	A long-term influence of inulin on KO mouse model for hyperuricemia	Improve intestinal barrier function, alleviate inflammatory state, and reduce serum UA levels, with a raised abundance of *Akkermansia* and *Ruminococcus*, and increased production of SCFAs.	(151)
2021	Chlorogenic acid (CGA)	CGA was orally administered to the corresponding hyperuricemia model mice for 19 days	Downregulation of XOD activities and the regulation of mRNA expression of UA excretory proteins	(152)
2021	Curcumin	The rat model of uric acid nephropathy intra-gastrically administered 200 mg/kg body weight curcumin daily for 8 weeks	Lower the levels of uremic toxins ameliorates inflammation and fibrosis in the kidneys by regulating the structure of intestinal flora and improving intestinal permeability	(153)
2019	*L.gasseri* PA3	A total of 45 samples from adults were conducted in an *in vitro* single-stage colonic fermentation	Reduce UA concentrations by weakening purine metabolism, increase the relative abundances of *Lactobacillus* (73.5%) and *Escherichia* (36.5%), and decrease *Bacteroides* and *Phascolarctobacterium*	(154)
2021	Lactic acid bacteria (LAB)	10 LAB strains isolated from human feces in a mouse model of PO- and hypoxanthine-induced hyperuricemia	Effectively downregulate serum UA concentrations and inhibited XOD activity; promote the production of SCFAs in the caecum and induce changes in the SCFA production-related taxa of the gut microbiota	(155)
2018	*Lactobacillus brevis* DM9218	Mice were fed with a normal diet, a high-fructose diet, or a high-fructose diet with DM9218	Decrease serum UA levels and hepatic xanthine oxidase activity in fructose-fed mice.; protect against high-fructose induced liver damage and retard UA accumulation by degrading inosine; enhance intestinal barrier function and reduce liver lipopolysaccharide	(156)
2019	Tuna meat oligopeptides (TMOP)	The mice in the LD-TMOP and HD-TMOP groups were gavage 50 mg·kg−1·d−1 and 300 mg·kg−1·d−1 TMOP for 8 weeks, respectively.	Inhibits the activation of NLRP3 inflammasome and TLR4/MyD88/NF-κB signaling pathways, suppressing the phosphorylation of p65-NF-κB. repair the intestinal epithelial barrier, reverse the gut microbiota dysbiosis and increase the production of short-chain fatty acids	(157)

Furthermore, a few studies (171, 172) recently have evaluated the effect of urate-lowering therapy with the synbiotic addition on the treatment of patients with gout. An addition of a synbiotic was demonstrated to have a more pronounced urate-lowering effect and was combined with a decrease in the level of CRP, IL-1β, IL-6, IL-8, IL-10, and TNFα (171, 172). Collectively, prebiotics and probiotics can maintain the balance of intestinal microbiota composition, inhibit XO activity and UA reabsorption, promote UA excretion, regulate intestinal epithelial cell proliferation, and relieve chronic inflammation to achieve the prevention and treatment of hyperuricemia and gout. However, we should notice the difference in UA metabolism between humans and experimental animals, such as rats; thus, additional clinical trials with large samples are needed to elucidate the potential of the probiotics in the prevention and treatment of patients with gout.

Besides, patients often have questions about dietary management of gout or hyperuricemia. Due to UA being a byproduct of fructose metabolism, fructose intake has gained increased attention in gout or hyperuricemia management. Fructose is the main component of sucrose and an important sweetener used in food production. It is found in many foods, including soft drinks, ketchup, barbecue (BBQ) sauce, fruits, etc.

Among them, the impact of SSBs on gout or hyperuricemia has received the greatest attention, and it is can be concluded that there was moderate certainty of evidence that SSBs have a deleterious effect on gout or hyperuricemia (65, 66).

Over 100 years ago, diets low in fructose as a mean to prevent gout has already been prescribed, and that sweeter fruit should not be taken was being suggested (173). However, natural fruit currently is considered to be healthy food, and the data with natural fruit effect on gout are mixed. To date, a number of studies have explored the relationship between fruit consumption and hyperuricemia. For instance, Tanya et al. explored the association between individual dietary components and the SUA level using a meta-analysis and indicated that cheese and non-citrus fruit intake were associated with lower SUA levels (66). Although a series of studies have reported a protective effect of fruit intake on gout (66, 174–177), one problem that cannot be ignored is that people who eat a lot of fruit usually eat a healthier diet with lower refined sugar content. Indeed, a prospective study (61) conducted in 2008 on 46,393 people without a history of gout showed that the multivariate relative risk of gout attacks for a daily serving of orange or apple for a month was significantly higher than the risk of less than one serving. And a meta-analysis that systematically explored the relation between fruit and incident gout and hyperuricemia, found that fruit intake was not associated with gout incident (58).

To explain the result of the paradox and to obtain a piece of advice on whether to eat fruit, several reasons should be considered. Firstly, the study design has affected the results. The measurement of the total fruit intake is obtained through retrospective questionnaire surveys or interviews in the above studies, resulting in an insufficient accuracy of the measurement. Secondly, the inconsistency of research in different countries may be related to latitude and longitude. The climate is a well-known factor to affects the sugar content of fruits. For example, as the fruit matures, the vitamin C content decreases and the sugar content increases (178). Thirdly, the effect of fruit on hyperuricemia may be far more complicated than we thought. Vitamin C has been reported to carry the ability to reduce serum UA concentrations (179, 180). Some types of cherries were demonstrated to reduce flares of gout (181, 182). Other crucial nutrients in fruits, including potassium, anthocyanins, catechins and flavonoids, could play an important role in lessening the pathological effects of UA and fructose (9).

Since the effect of fruit on hyperuricemia is so complicated, what about fruit juice? It is suggested that patients with gout or hyperuricemia should avoid food high in HFCS, like soft drinks. But whether the fructose-containing fruit juice often compared to soft drinks at the same amounts, has a negative impact on gout or not? To date, few studies have evaluated the effect of fruit juice on UA metabolism and produced some interesting results. One of the interesting results is that consumption of orange juice was related to a higher incidence of gout, but apple juice, grapefruit juice, tomato juice and others were not (62). Another data from a large-sample prospective cohort study, however, has explored the effect of orange juice and other fruit juices and found that both orange juice and total fruit juice were associated with the risk of gout (61). And in a meta-analysis study, it was found that fruit juice is related to a 77% increase in gout (68). But in the NHANES (2001-02), intake of fruit juice, which contains a lot of naturally occurring sugars, was revealed not to be related to SUA (64). It is postulated that fructose concentration in different juice may be the main reason.

Taken together, though the data about the effect of natural fruit and fruit juice on the risk of hyperuricemia and gout are somehow mixed, it is recommended to reduce the intake of refined sugars and fruit juices in the management of gout patients, and encourage the intake of natural fruits, especially cherries, non-citrus fruit, and less ripe fruit in which fructose content is low. In addition, probiotics in the prevention or treatment of hyperuricemia or gout is still in its infancy. Regulating the intestinal flora and maintaining intestinal homeostasis via the use of probiotics and prebiotics or even fecal transplantation may be a new intestinal microecological treatment method for hyperuricemia and gout. If these findings are replicated in future randomized controlled trials, are substantial.

## Conclusions and Future Perspectives

A recent alarming increase in the hyperuricemia subjects with associated gout and other detrimental metabolic diseases has sparked extensive research in the field. This review provides a pivotal role of the high-dose fructose intake and gut microbiota in the pathogenesis of hyperuricemia and gout. Excessive fructose (especially in refined sugar and fruit juice) intake alters the gut microbiota composition and impairs intestinal barriers function through a series of inflammatory reactions, which play key roles in the pathogenesis of hyperuricemia and gout. In turn, the inflammatory reactions triggered by high-dose fructose intake also have a significant impact on the shaping and maintaining of the intestinal flora homeostasis, which is critical for the catabolism of purines and UA. The results described suggest that it might be safe, effective and side-effect-free approach for the prevention or treatment of hyperuricemia and gout to limit specific fructose intake and improve the composition of gut microbiota or target characteristic metabolites of beneficial bacteria via the use of probiotics or prebiotics. Nevertheless, the mechanism by which fructose intake interacts with the gut microbiota has not been fully elucidated. Current research evidence mostly comes from animal experiments, the differences between humans and experimental animals (such as rats) limit the extrapolation of the results. Therefore, further clinical trials are needed to clarify its specific mechanism. In addition, some probiotics or prebiotics that have been shown useful in the treatment of hyperuricemia and gout are mixed extracts, and the mechanisms by which they mediate resistance to hyperuricemia and gout involve multiple targets and multiple pathways. Further identification of the specific components involved in lowering UA is needed to provide a broader range of selection of effective treatments for hyperuricemia and gout in the future. And whether the role of the gut microbiota is necessary or synergistic needs to be further clarified through integrated multiomic approaches. Also, the safety and effects of the application of probiotics in clinical use need verification. Besides, more prospective studies are necessary in the future to evaluate intake of various fructose-containing food sources in different populations and their relationship to gout and hyperuricemia. These future directions will help identify the extent to which our foods and probiotics regulate the risk of hyperuricemia and gout, which will further inform health care professionals, policymakers, and help develop improved dietary guidelines for the prevention and management of gout and hyperuricemia.

## Author Contributions

D-qY: ideas. X-yF: ideas and writing the initial draft. L-wQ, H-fC, PG, and QZ: literature collection. R-xL, Y-gF, B-zL, and H-fP: writing—review and editing. All authors contributed to the article and approved the submitted version.

## Funding

This study was supported by the National Natural Science Foundation of China (82073652).

## Conflict of Interest

The authors declare that the research was conducted in the absence of any commercial or financial relationships that could be construed as a potential conflict of interest.

## Publisher's Note

All claims expressed in this article are solely those of the authors and do not necessarily represent those of their affiliated organizations, or those of the publisher, the editors and the reviewers. Any product that may be evaluated in this article, or claim that may be made by its manufacturer, is not guaranteed or endorsed by the publisher.
